# Functional Involvement of TANK-Binding Kinase 1 in the MyD88-Dependent NF-*κ*B Pathway Through Syk

**DOI:** 10.1155/2024/8634515

**Published:** 2024-10-26

**Authors:** Han Gyung Kim, Ji Hye Kim, Tao Yu, Jae Youl Cho

**Affiliations:** ^1^Department of Integrative Biotechnology and Biomedical Institute for Convergence at SKKU (BICS), Sungkyunkwan University, Suwon 16419, Republic of Korea; ^2^Institute for Translational Medicine, The Affiliated Hospital of Qingdao University, Qingdao 266000, China

**Keywords:** inflammation, macrophages, NF-*κ*B, Syk, TBK1

## Abstract

Inflammation is a vital immune defense mechanism regulated by Toll-like receptors (TLRs) and the nuclear factor-kappa B (NF-*κ*B) pathway. TANK-binding kinase 1 (TBK1) is central to immunity and inflammation and influences antiviral responses and cellular processes. However, the precise role of TBK1 in modulating the NF-*κ*B pathway through interactions with other proteins, such as spleen tyrosine kinase (Syk), remains poorly understood. As dysregulation of TBK1 and NF-*κ*B can lead to a variety of diseases, they are important therapeutic targets. In this work, inflammatory processes involving the TBK1-Syk-NF-*κ*B pathway were elucidated using lipopolysaccharide (LPS)-induced macrophages; human embryonic kidney 293 (HEK293) cells overexpressing MyD88, TBK1, and Syk proteins and their mutants; and real-time polymerase chain reaction (PCR), immunoblotting analyses, and kinase assays. TBK1 was activated in LPS-, poly I:C-, and Pam3CSK-stimulated macrophages. Transcript levels of *TNF*, *NOS2*, and *IL1B* were increased in cells overexpressing TBK1 but not in cells overexpressing TBK1 K38A. The transcription of *TNF*, *NOS2*, and *IL1B* and NF-*κ*B luciferase activity were inhibited by silencing TBK1 in LPS-stimulated RAW264.7 cells and MyD88-transfected HEK293 cells. Syk was the key mediator of the TBK1-dependent NF-*κ*B pathway and bound directly to the coiled coil domain of TBK1, which was necessary to activate Syk and the Syk-p85 pathway. This research advances the understanding of the role of TBK1 in NF-*κ*B signaling, emphasizing Syk as a key mediator. The interaction between TBK1 and Syk has potential for precise immune modulation that can be applied to treat immune-related disorders.

## 1. Introduction

Inflammation is a dynamic and complex biological response that serves as a fundamental component of the immune system's defense mechanisms. It is a regulated response that helps protect the body from infections, injuries, tissue damage, and pathogens [1]. This process involves a series of coordinated cellular and molecular events mediated by immune cells, cytokines, and signaling pathways [2]. Central to this regulation are Toll-like receptors (TLRs), a group of pattern recognition receptors that detect conserved molecular patterns commonly associated with pathogens [3, 4]. TLRs play a critical role in initiating immune responses and are a key element of the innate immune system [5]. The precise regulation of inflammation is essential, as dysregulated inflammatory responses can lead to a broad range of health issues, including autoimmune disease and chronic inflammatory disorders. Therefore, understanding of the critical proteins involved in the TLR-mediated pathway is of critical importance.

TANK-binding kinase 1 (TBK1) is a pivotal serine/threonine protein kinase that is essential for several cellular functions. TBK1 is a key regulator in signaling pathways that trigger immune reactions against viral threats [6–9]. In addition to its established antiviral role, TBK1 influences a variety of cellular processes, including cell proliferation, apoptosis, autophagy, and antitumor immunity [10, 11]. Dysregulation of TBK1 activity has been implicated in autoimmune disorders, neurodegenerative diseases, and tumorigenesis [10, 12, 13]. Moreover, TBK1 is closely tied to the inflammatory signaling pathway and affects the responses to inflammatory stimuli [6]. It operates predominantly in the stimulator of interferon genes (STING) and TLR pathways, which regulate cellular inflammation and immunity [14, 15]. However, the role and the partners of TBK1 in the TLR-mediated pathway are not clear.

Spleen tyrosine kinase (Syk) plays a critical role in inflammatory responses in macrophages [16] and is a key regulator for the signaling cascade in the MyD88-dependent TLR pathway [17]. Above all, the activation of Syk leads to subsequent activation of p85 and the nuclear factor-kappa B (NF-*κ*B) pathway, which plays a critical role in the inflammatory signals in macrophages [18, 19]. The NF-*κ*B transcription factor complex consists of many subunits, including p50, p65, and IκB proteins [20]. Syk in the NF-*κ*B pathway is essential for maintaining immune homeostasis and defending against pathogens. However, dysregulation of this pathway can lead to chronic inflammation and contribute to the development of a range of diseases, including autoimmune disorders, chronic inflammatory conditions, and cancer [21]. Therefore, targeting Syk can be a strategy to block many inflammatory diseases.

Despite previous studies of TBK1, Syk, and the NF-*κ*B pathway, the interaction between TBK1 and Syk in activation of the NF-*κ*B pathway during an inflammatory response is not clearly understood. In this study, we focus on the role of TBK1 in the NF-*κ*B pathway and upstream adopter molecules, including MyD88 and TRIF. We also identify the specific binding domain of TBK1 and Syk. Our findings suggest that the interaction of TBK1 and Syk is a key event in the activation of the NF-*κ*B pathway in an inflammatory response.

## 2. Materials and Methods

### 2.1. Materials

Roswell Park Memorial Institute 1640 (RPMI 1640) cell culture medium, Dulbecco's modified Eagle's medium (DMEM), fetal bovine serum (FBS), penicillin/streptomycin (P/S) 100× solution, and phosphate-buffered saline (PBS) were purchased from Hyclone. Lipofectamine 3000, TRIzol reagent, MuLV reverse transcriptase, pfu DNA polymerase, competent DH5*α* cells, polyvinylidene difluoride (PVDF) membranes, and enhanced chemiluminescence reagent were purchased from Thermo Fisher Scientific (Waltham, MA, USA). Piceatannol, BAY 61-3606 [22], PP2, BX795, Wortmannin, LY294002, BAY-11-7082, bovine serum albumin (BSA), lipopolysaccharide (LPS), poly I:C, Pam3CSK, luciferin, and sodium dodecyl sulfate (SDS) were purchased from Sigma Chemical Co. (St. Louis, MO, USA). The antibodies specific for phosphor (p)-TBK1 (CST5483), TBK1 (CST3013), p-Syk (CST 2711), Syk (CST 2712), p-p85 (CST 4228), p85 (CST 4292), p-IκB*α* (CST 9246), IκB*α* (CST 9247), Flag (CST 8146), Myc (CST 2276), CFP (CST 2956), and *β*-actin (CST 4967) used for western blot analyses, immunoprecipitation, and immunofluorescence staining were purchased from Cell Signaling Technology (Beverly, MA, USA). The siRNAs of Syk (5′-CGCUCUUAAAGAUGAGUUATT-3′) and TBK1 (5′-GCUGUUGUCUGACAUUCUATT-3′) used for gene silencing were designed and synthesized at Genolution (Seoul, South Korea).

### 2.2. Cell Cultures

RAW264.7 cells were cultured in RPMI 1640, and human embryonic kidney 293 (HEK293) cells were cultured in DMEM with 10% FBS and P/S. Cell lines were purchased from American Type Culture Collection (ATCC) (Manassas, VA, USA) and incubated at 37°C in a humidified atmosphere containing 5% CO_2_. All cell lines were tested for mycoplasma contamination using a BioMycoX mycoplasma polymerase chain reaction (PCR) detection kit (CellSafe, Seoul, South Korea).

### 2.3. Protein Isolation and Western Blotting

Cultured cells (5 × 10^6^ cells/mL of RAW264.7 or HEK293 cells) were lysed using a buffer composed of 50 mM Tris–hydrochloride (Tris–HCl) pH 7.5, 20 mM sodium fluoride (NaF), 25 mM *β*-glycerol phosphate pH 7.5, 120 mM sodium chloride (NaCl), 2% NP-40, and protease inhibitor cocktail. The concentration of isolated protein was measured by Bio-Rad protein assay dye reagent concentrate (50000006, Bio-Rad). Subsequently, the protein levels were assessed with western blotting. Proteins were separated on 10% or 12% SDS–polyacrylamide gels, transferred onto PVDF membranes, and then blocked in Tris-buffered saline containing 3% BSA. Finally, the results were visualized using EzWestLumi plus (ATTO, Tokyo, Japan).

### 2.4. RNA Precipitation and Real-Time PCR

The extraction of total RNA was carried out utilizing TRIzol (Gibco BRL, Grand Island, NY, USA). Subsequently, complementary DNA (cDNA) was synthesized using MMLV RTase (SuperBio, Daejeon, South Korea), followed by real-time PCR. The primers utilized for real-time PCR are detailed in Table 1, all of which were procured from Macrogen, Inc. (Seoul, South Korea).

### 2.5. Construction of Expression Vectors

A Flag-tagged wild-type (WT) TBK1 construct (Flag-TBK1-WT) and a Myc-tagged WT Syk construct were generated by amplification through a standard culture method employing competent *Escherichia coli* (DH5*α*). Additionally, pCMV-Flag-TBK1 and pCMV-Myc-Syk domain deletion mutants utilized in this study were created using a QuikChange Lightning Site-Directed Mutagenesis Kit. To verify the sequences of all constructs, a BigDye Terminator v3.1 Cycle Sequencing Kit (Biosystems) was employed, which was sourced from Macrogen, Inc. (Seoul, South Korea).

### 2.6. Luciferase Reporter Gene Assay

HEK293 cells (1 × 10^6^ cells/mL) were transfected with plasmids containing NF-*κ*B-Luc (E8491, Promega) or AP-1 (E4111, Promega) and *β*-galactosidase, along with cotransfection of plasmid constructs containing TBK1-WT, TBK1-K38A, MyD88, and TRIF, utilizing the polyethyleneimine method in a 24-well plate. The experiments were conducted 48 h after transfection. Luciferase assays were carried out using a luciferase assay system (Promega, Madison, WI, USA) in accordance with the manufacturer's instructions.

### 2.7. Immunoprecipitation

Cell lysates containing equal amounts of protein (500 μg for exogenous protein, 1000 μg for endogenous protein) were incubated overnight at 4°C with 5 μL of either anti-Myc, anti-Flag, or anti-CFP antibodies. The resulting proteins and antibody complexes were then combined with 20 μL of protein A or G Sepharose 4 Fast Flow Beads (50% v/v, GE Healthcare Life Sciences, Marlborough, MA, USA). Subsequently, both whole cell lysates and immunoprecipitates were subjected to analysis by immunoblotting. Mouse TrueBlot ULTRA, Anti-Mouse IgG HRP and Rabbit TrueBlot, Anti-Rabbit IgG HRP (Rockland Immunochemicals, Pottstown, PA, USA) were used as secondary antibodies to prevent immunoglobulin G (IgG)-heavy chain blotting [23].

### 2.8. In Vitro Protein Kinase Assay

Purified TBK1, TBK1-K38A, TBK1-ΔCC, TBK1-ΔULD, and Syk were used as enzyme sources for the in vitro kinase assay, and purified p85 was used as the substrate of the in vitro kinase assay. The enzymes and substrates were mixed with the reaction buffer in a final reaction volume of 25 μL. The reaction was initiated by the addition of magnesium and adenosine triphosphate (ATP) and incubated for 1 h at 25°C [24]. The reaction was terminated by adding 5 mL of a 3% phosphoric acid solution. Phosphorylation of the substrates was detected by western blotting.

### 2.9. Confocal Microscopy

For confocal microscopy, 2 × 10^5^ cells were seeded in 12-well plates with sterile coverslips. After transfecting the DNA, the cells were washed twice with 1 mL of PBS. Subsequently, the cells were fixed with 3.7% paraformaldehyde, washed three times with PBS, and then blocked in 1% BSA/PBS-T. The polymerized actin, nuclei, and specific target proteins were stained with rhodamine phalloidin (Invitrogen), Hoechst (1:1000), or the appropriate antibodies. Coverslips were mounted onto slides using fluorescent mounting medium (DakoCytomation, Carpentaria, CA, USA). The cells were then viewed with confocal microscopy (LSM800, Zeiss) [23].

### 2.10. Statistics and Reproducibility

Unless otherwise specified, all data are expressed as the mean ± standard deviation (SD) of a minimum of three independent experiments. Statistical assessments were performed using the Kruskal–Wallis test followed by the Mann–Whitney test. All *p* values ≤0.05 were considered statistically significant. The data analysis was conducted using GraphPad Prism version 8.0 (GraphPad Software).

## 3. Results

### 3.1. TBK1 Induces an Inflammatory Response Through a TLR-Dependent Pathway

**Figure 1:**
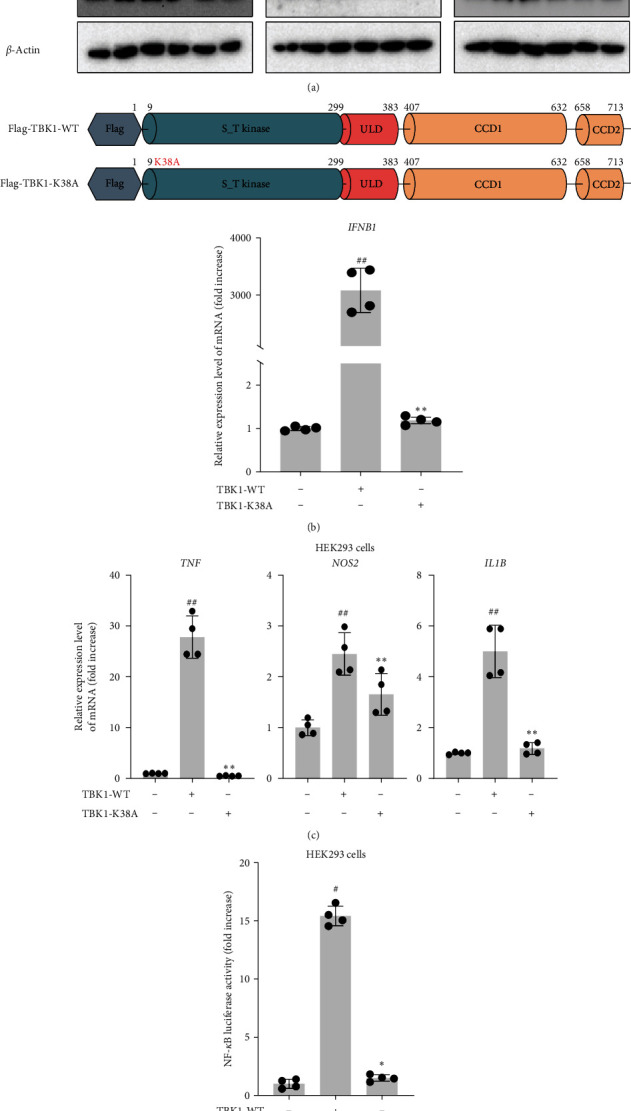
The functional role of TANK-binding kinase 1 (TBK1) in Toll-like receptor (TLR)–dependent pathways: (a) TBK1 and p-TBK1 were detected by immunoblot analyses of RAW264.7 cells treated with lipopolysaccharide (LPS) (1 μg/mL), poly I:C (200 μg/mL), or Pam3CSK (1 μg/mL) for the indicated times; (b, c) human embryonic kidney 293 (HEK293) cells were transfected with TBK1 WT and TBK1-K38A, and the expression of IL1B, TNF, NOS2, and IFNB1 was measured using quantitative real-time polymerase chain reaction (PCR); and (d) HEK293 cells were transfected with nuclear factor-kappa B (NF-*κ*B) and TBK1 WT or TBK1-K38A for 24 h. The data are presented as the mean ± standard deviation (SD). Statistical significance was assessed using the Kruskal–Wallis test followed by the Mann–Whitney test. ^#^*p* < 0.05 and ^##^*p* < 0.01 versus control group; *⁣*^*∗*^*p* < 0.05 and *⁣*^*∗∗*^*p* < 0.01 versus TBK1-WT-overexpressing group. WT, wild type.

We investigated the activators of TBK1 during an inflammatory response by assessing TBK1 phosphorylation via western blot analyses. The results demonstrate that TBK1 phosphorylation was induced in response to stimulation with LPS (1 μg/mL), poly I:C (200 μg/mL), or Pam3CSK (1 μg/mL). Therefore, TBK1 is a crucial modulator in the TLR2-, TLR3-, and TLR4-dependent pathways (Figure 1a). Moreover, we tested HEK293 cells overexpressing TBK1-WT or TBK1-K38A (the inactivated form of TBK1) to examine their effects on cytokine expression [25]. Following the overexpression of TBK1-WT or TBK1-K38A, mRNA isolation and real-time PCR analysis were performed to evaluate the expression of inflammatory cytokines. Notably, in HEK293 cells, the overexpression of TBK1-WT led to an increase in IFNB1 expression, whereas the overexpression of TBK1-K38A resulted in a decrease of IFNB1 (Figure 1b). Additionally, the expression levels of *IL1B*, *TNF*, and *NOS2* were upregulated in cells overexpressing TBK1-WT, and their expression was not induced in cells overexpressing TBK1-K38A (Figure 1c). Moreover, to assess alterations in the transcriptional factors associated with the inflammatory response, including NF-*κ*B in the TBK1-dependent pathway, reporter gene assays were performed with TBK1-WT- and TBK1-K38A-transfected HEK293 cells. The luciferase activity of NF-*κ*B was induced by the overexpression of TBK1-WT but not by the overexpression of TBK1-K38A (Figure 1d).

### 3.2. TBK1 Has a Critical Role in the MyD88-Dependent NF-*κ*B Pathway

**Figure 2:**
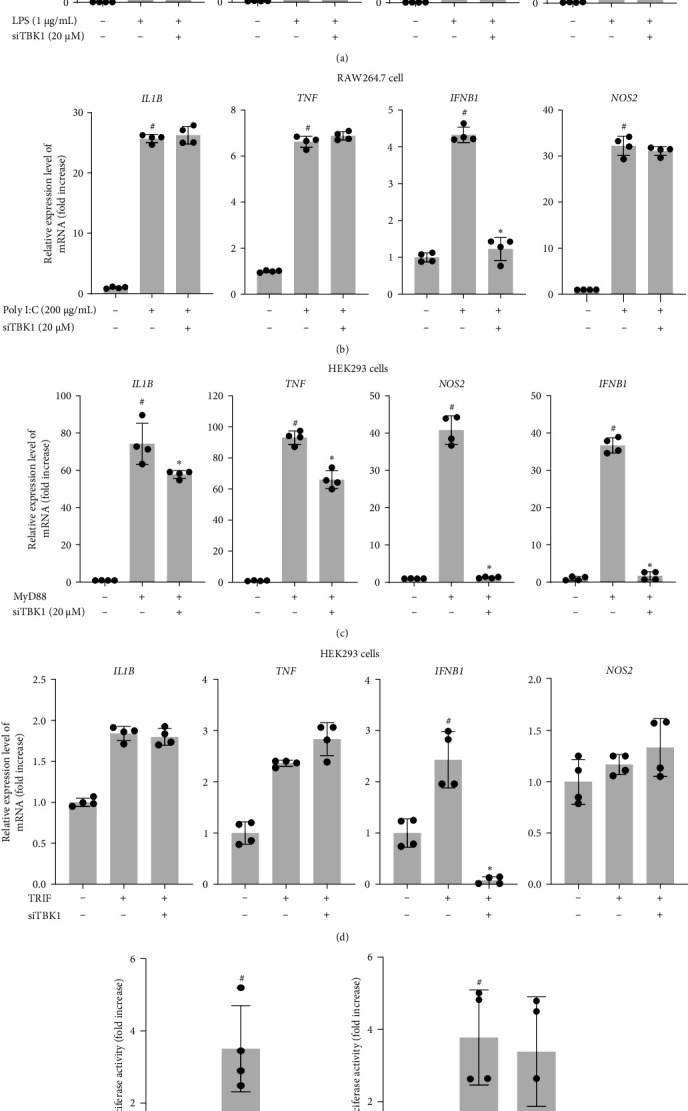
TANK-binding kinase 1 (TBK1) silencing inhibits MyD88-dependent signaling pathways: (a, b) RAW264.7 cells were transfected with siTBK1 (20 μM) or siNC (negative control) for 48 h and then stimulated with (a) lipopolysaccharide (LPS) (1 μg/mL) or (b) poly I:C (200 μg/mL). Then, the mRNA expression of IL1B, TNF, IFNB1, and NOS2 was measured using quantitative real-time polymerase chain reaction (PCR) (*n* = 4). (c, d) Human embryonic kidney 293 (HEK293) cells were transfected with siTBK1 (20 μM) or siNC for 48 h and then transfected with (c) MyD88 or (d) TRIF. Then, the mRNA expression of IL1B, TNF, IFNB1, and NOS2 was measured using quantitative real-time PCR (*n* = 4). (e, f) HEK293 cells were transfected with siTBK1 (20 μM) or siNC for 48 h and cotransfected with the nuclear factor-kappa B (NF-*κ*B)-luciferase reporter gene and (e) MyD88 or (f) TRIF for 24 h. The luciferase activity of NF-*κ*B was detected with a luminometer and normalized to B-galactosidase activity (*n* = 4). The data are presented as the mean ± standard deviation (SD). Statistical significance was assessed with the Kruskal–Wallis test followed by the Mann–Whitney test. ^#^*p* < 0.05 and ^##^*p* < 0.01 versus control group; *⁣*^*∗*^*p* < 0.05 and *⁣*^*∗∗*^*p* < 0.01 versus LPS- or poly I:C-treated or MyD88- or TRIF-transfected group. IFN-*β*, interferon beta; IL-1*β*, interleukin-1 beta; iNOS, inducible nitric oxide synthase; TNF-*α*, tumor necrosis factor alpha.

To ascertain the role of TBK1 in the MyD88-dependent NF-*κ*B pathway, the expression of cytokines induced during an inflammatory response was measured with real-time PCR. Inflammatory cytokines (IL1B, TNF, IFNB1, and NOS2) were significantly decreased in RAW264.7 cells treated with siTBK1 under LPS stimulation (Figure 2a). Poly(I:C)-stimulated siTBK1-expressing cells decreased their expression of IFNB1, but IL1B, TNF, and NOS2 expression was not altered (Figure 2b). LPS induced the MyD88- and TRIF-dependent signaling pathways, but poly I:C activated only the TRIF-dependent signaling pathway [26]. Taken together, these data show that TBK1 regulates the MyD88-dependent inflammatory response.

To identify the functional role of TBK1 in the MyD88-dependent TLR signaling pathway, the mRNA expression of inflammatory cytokines (IL1B, TNF, IFNB1, and NOS2) was examined using real-time PCR. As shown in Figure 2c,d, IL1B, TNF, and NOS2 expression was reduced by silencing TBK1 only in RAW264.7 cells overexpressing MyD88, not TRIF. Furthermore, NF-*κ*B-mediated luciferase reporter gene activity was examined in HEK293 cells transfected with MyD88, TRIF, or siTBK1. Silencing of TBK1 strongly inhibited NF-*κ*B-mediated luciferase reporter gene activity compared with HEK293 cells stimulated by MyD88 but not by TRIF (Figure 2e). The NF-*κ*B-related gene expression of IL1B, TNF, and NOS2 and the luciferase activity of NF-*κ*B induced by poly I:C or TRIF were not regulated by TBK1 (Figure 2a–d). However, the expression of IFNB1 differentially induced by LPS, poly I:C, MyD88, or TRIF was regulated by siTBK1. This difference suggested that TBK1 plays a significant role in the MyD88-dependent NF-*κ*B pathway for increasing mRNA levels of IL1B, TNF, and NOS2. Also, the difference of expression of IFNB1 between MyD88 (or LPS)-dependent and TRIF (or poly I:C)-dependent pathways reveal the important role of the MyD88 pathway as a major signaling inducer in IFNB expression.

### 3.3. Syk Is the Key Molecule in the MyD88-/TBK1-Mediated NF-*κ*B Signaling Pathway

**Figure 3:**
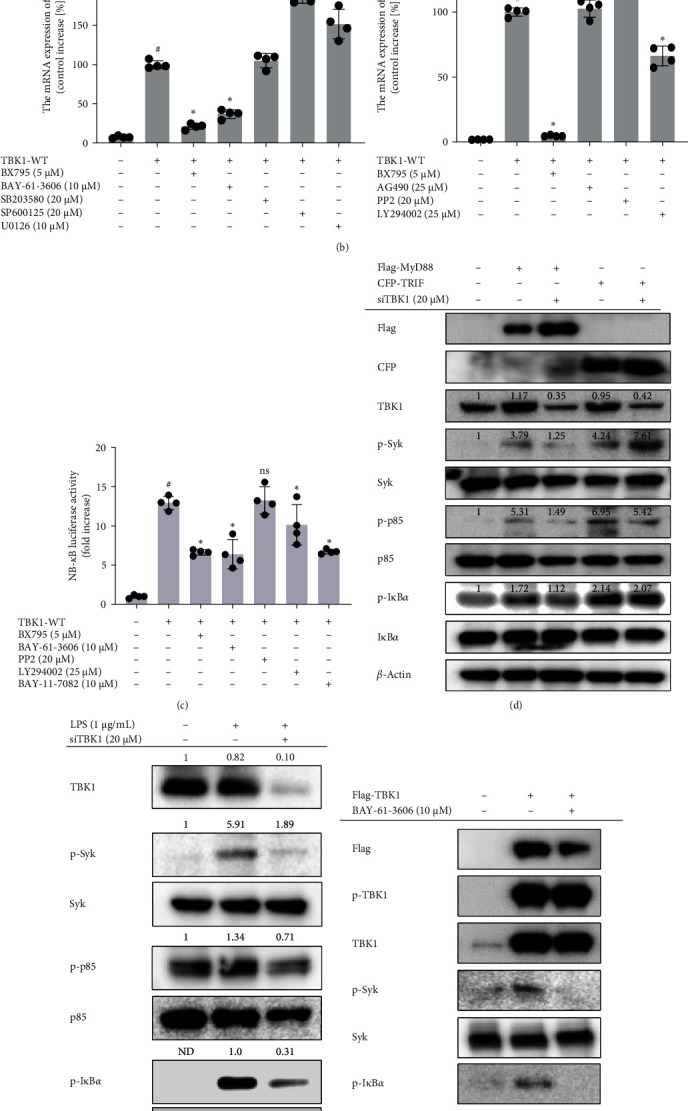
TANK-binding kinase 1 (TBK1) mediates the nuclear factor-kappa B (NF-*κ*B) signaling pathway. (a) Chemical structures of BX795 (TBK1 inhibitor), BAY-11-7082 (NF-*κ*B inhibitor), LY294002 (PI3K inhibitor), and BAY-61-3606 (Syk inhibitor). (b) HEK293 cells were transfected with TBK1 for 24 h and then separately treated with BX795, BAY-61-3606, SB203580 (p38 inhibitor), SP600125 (JNK inhibitor), U0126 (ERK inhibitor), AG490 (JAK inhibitor), PP2 (Src inhibitor), LY294002, or Wortmannin (PI3K inhibitor) for 24 h. The mRNA expression of tumor necrosis factor (TNF) was determined using real-time polymerase chain reaction (PCR). (c) Human embryonic kidney 293 (HEK293) cells were cotransfected with TBK1 and NF-*κ*B-Luc constructs for 24 h and then treated with BX795, BAY-61-3606, PP2, Wortmannin, LY294002, or BAY-11-7082 for 24 h. Luciferase activity was measured using a luminometer and normalized to *β*-gal values. (d) HEK293 cells were cotransfected with siTBK1 and MyD88 or TRIF for 48 h. (e) RAW264.7 cells were transfected with siTBK1 for 48 h and then stimulated with lipopolysaccharide (LPS) (1 μg/mL) for 5 min. (f) HEK293 cells overexpressing TBK1 were incubated with BAY-61-3606 (10 μM) for 24 h. (e, f) The expression of Flag, CFP, TBK1, and *β*-actin and the phosphorylated and total levels of Syk, p85, and IκB*α* were detected in whole lysates using western blotting. ImageJ was used to measure and quantify band intensity. The data are shown as the mean ± standard deviation (SD), *n* = 4. ^#^*p* < 0.05 and ^##^*p* < 0.01 versus control group; *⁣*^*∗*^*p* < 0.05 and *⁣*^*∗∗*^*p* < 0.01 versus TBK1-WT transfected group. ND, not detected; ns, not significant.

To determine which key player in the NF-*κ*B signaling pathway is induced by TBK1, TNF was evaluated in TBK1-overexpressing HEK293 cells treated with kinase inhibitors. The mRNA expression of TNF was inhibited by BX795 (TBK1 inhibitor), LY294002 (PI3K inhibitor), and BAY-61-3606 (Syk inhibitor) (Figure 3a,b). Additionally, NF-*κ*B-Luc reporter activity was inhibited by BX795, BAY-61-3606, and BAY-11-7082 in TBK1-activated HEK293 cells (Figure 3c). To examine the activation of Syk, p85, and IκB*α*, their phosphorylation was evaluated in HEK293 cells stimulated by MyD88 and TRIF. The phosphorylation of Syk, p85, and IκB*α* induced by MyD88 was reduced by TBK1 silencing (Figure 3d). However, the activation of Syk, p85, and IκB*α* stimulated by TRIF showed no suppression in HEK293 cells treated with siTBK1 (Figure 3d). Moreover, the silencing of TBK1 was identified in RAW264.7 cells treated with LPS. Phosphorylation of Syk, p85, and IκB*α* was reduced in RAW264.7 cells treated with LPS and then siTBK1 (Figure 3e). To determine whether the IκB*α* activation induced by TBK1 depended on Syk regulation, phospho-IκB*α* was evaluated with immunoblot analysis in HEK293 cells transfected with TBK1 and treated with BAY-61-3606, a selective Syk inhibitor [27]. The TBK1-induced phosphorylation of IκB*α* and Syk was inhibited by BAY-61-3606 without affecting the phosphorylation of TBK1 (Figure 3f). These results indicate that the MyD88-/TBK1-dependent NF-*κ*B pathway is regulated by Syk.

### 3.4. TBK1 Mediates the NF-*κ*B Signaling Pathway by Regulating Syk Kinase Activity

**Figure 4:**
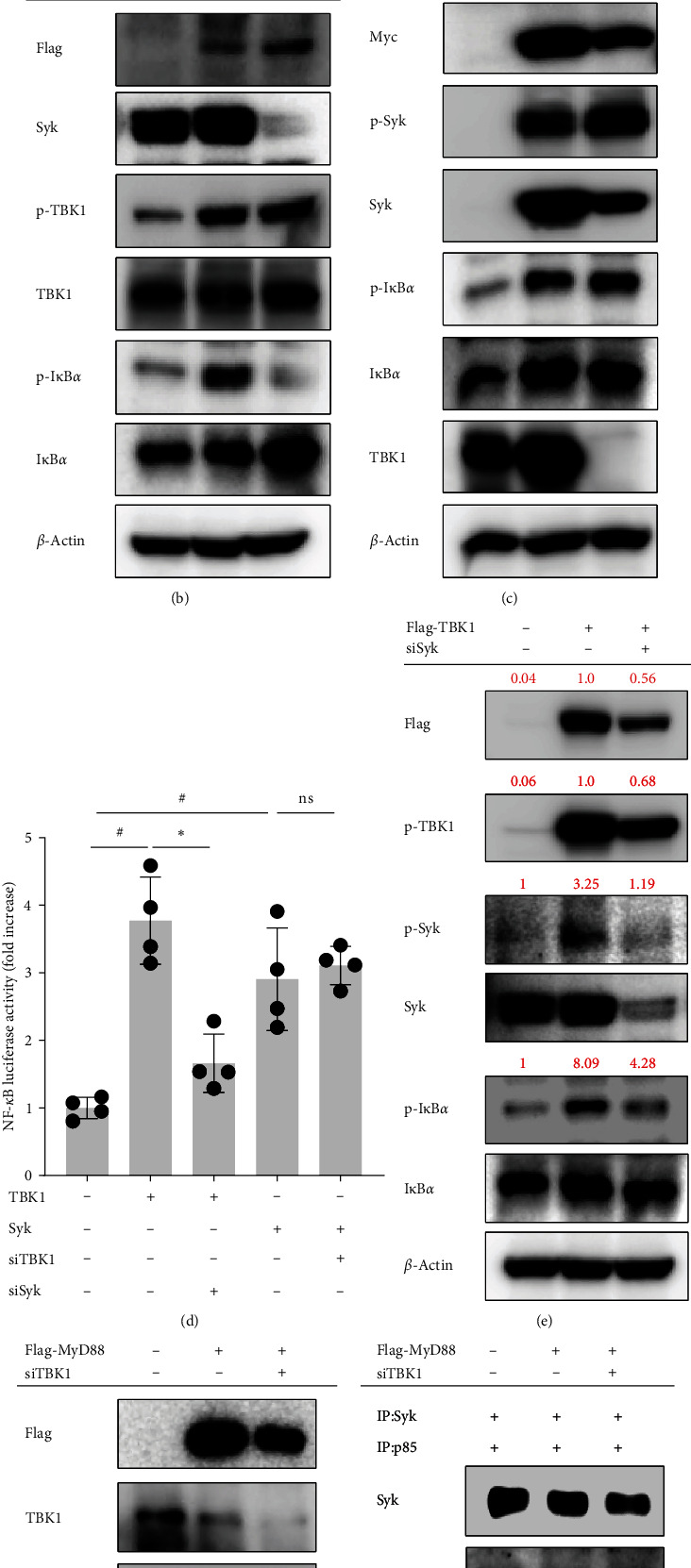
TANK-binding kinase 1 (TBK1) mediates the nuclear factor-kappa B (NF-*κ*B) signaling pathway by regulating Syk kinase activity. (a) RAW264.7 cells were treated with lipopolysaccharide (LPS) (1 μg/mL), poly I:C (200 μg/mL), or Pam3CSK (1 μg/mL) for the indicated times. Phospho- and total Syk were evaluated in whole cell lysates using western blot analyses. (b–d) RAW264.7 cells were cotransfected with MyD88, Syk, or TBK1 and siTBK1 or siSyk for 48 h. The phosphorylated and total levels of Syk, TBK1, and IκB*α* were detected using western blotting. *β*-Actin was used as a loading control. (e) Human embryonic kidney 293 (HEK293) cells were cotransfected with TBK1 or Syk, siTBK1 or siSyk, and NF-*κ*B-Luc constructs for 48 h. Then, NF-*κ*B-mediated luciferase activity was measured using a luminometer and normalized to *β*-gal values. (f) Kinase assays were performed using immunoprecipitated Syk and p85 in MyD88- and siTBK1-transfected HEK293 cells as enzyme and substrate sources, respectively. Flag, TBK1, Syk, p85, phospho-p85, and *β*-actin were detected by western blotting. The data are shown as the mean ± standard deviation (SD), *n* = 4. ^#^*p* < 0.05 and ^##^*p* < 0.01 versus control group; *⁣*^*∗∗*^*p* < 0.01 versus TBK1- or Syk-transfected group. ns, nonsignificant; TLR, Toll-like receptor.

We next investigated whether Syk activation occurs downstream of the MyD88/TBK1 pathway. Our previous findings substantiated the involvement of Syk in the activation of MyD88-IκB*α*, emphasizing its role in the macrophage inflammatory response rather than its association with TRIF [28]. To assess Syk activation in activated macrophages, we examined the phosphorylation of Syk in RAW264.7 cells that had been stimulated with LPS, poly I:C, or Pam3CSK. Syk was phosphorylated at 1 min in LPS- and Pam3CSK-stimulated RAW264.7 cells, while no significant alteration in Syk phosphorylation was observed in RAW264.7 cells stimulated with poly I:C (Figure 4a). In HEK293 cells stimulated with MyD88, Syk silencing inhibited IκB*α* phosphorylation but caused no discernible alteration in phospho-TBK1 levels (Figure 4b). In HEK293 cells, neither Syk overexpression nor siTBK1 treatment had an effect on IκB*α* or Syk phosphorylation (Figure 4c) or NF-*κ*B-mediated luciferase activity (Figure 4d). However, silencing Syk in TBK1-overexpressing HEK293 cells notably inhibited IκB*α* phosphorylation without reducing the phosphorylation of Flag-TBK1 (Figure 4e) or NF-*κ*B-mediated luciferase activity (Figure 4d). Subsequently, we conducted an in vitro kinase assay to elucidate the effects of TBK1 inhibition during Syk activation. In the presence of MyD88-induced Syk activation, we found concurrent phosphorylation of p85. Notably, treatment with siTBK1 led to a pronounced inhibition of p85 phosphorylation (Figure 4f). These results show that MyD88/TBK1 is an upstream activator of Syk in macrophages that leads to NF-*κ*B pathway activation.

### 3.5. TBK1 Interacts With Syk in Macrophages

**Figure 5:**
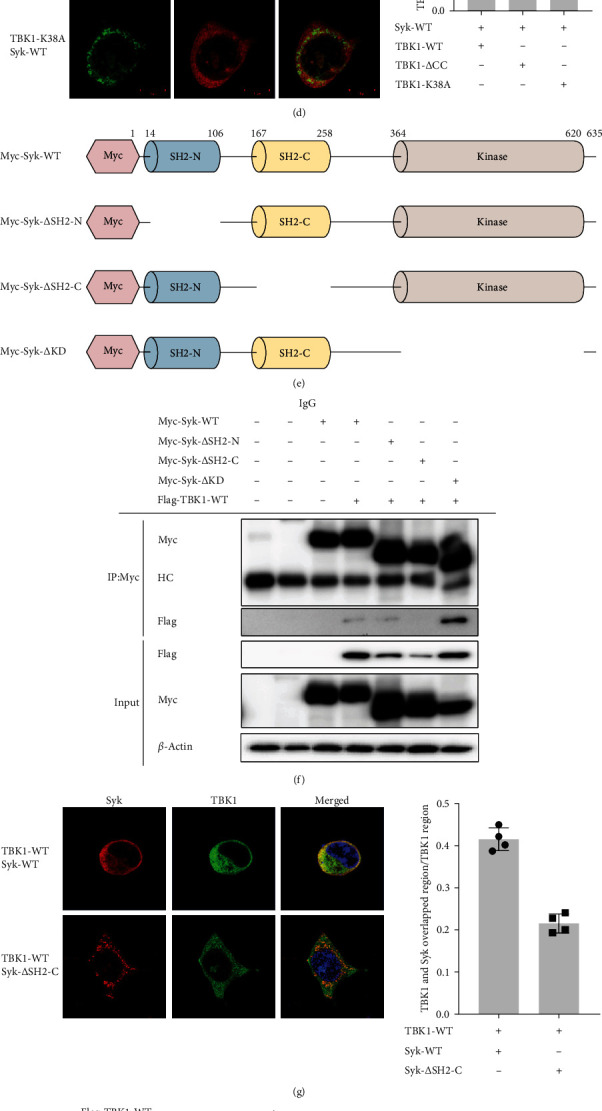
Interaction domain of TANK-binding kinase 1 (TBK1) and Syk. (a) Human embryonic kidney 293 (HEK293) cells overexpressing Flag-TBK1-WT, Flag-TBK1-K38A, or Myc-Syk-WT were lysed for an immunoprecipitation assay. Flag, Myc, and phospho-Syk levels were measured by western blotting of whole cell lysates (input), or immunoprecipitants obtained from whole cell lysates of the HEK293 cells were measured by anti-Flag (IP: Flag). (b) Diagram of TBK1 and its mutants (wild type [WT], coiled coil domain [ΔCC], ubiquitin-like domain [ΔULD], and kinase domain [ΔKD]). (c) HEK293 cells overexpressing Flag-TBK1-WT, Flag-TBK1-ΔCC, Flag-TBK1-ΔULD, or Flag-TBK1-ΔKD with Myc-Syk-WT were lysed for an immunoprecipitation assay. Flag and Myc levels were measured by western blotting using whole cell lysates (input), or immunoprecipitants obtained from whole cell lysates of the HEK293 cells were measured by anti-Flag (IP: Flag). (d) HEK293 cells were transfected with Myc-Syk-WT and Flag-TBK1-WT, Flag-TBK1-ΔCC, Flag-TBK1-ΔULD, or Flag-TBK1-ΔKD for 24 h and then permeabilized and fixed for confocal analysis. Alexa Fluor 488 and 568 were used to recognize TBK1 (green) and Syk (red), respectively. (e) Diagram of Syk and its mutants (WT, ΔSH2-N [SRC homology domain 2N-terminal], ΔSH2-C [SRC homology domain 2C-terminal], and ΔKD). (f) HEK293 cells overexpressed with Flag-TBK1-WT and Myc-Syk-WT, Myc-Syk-ΔSH2-N, Myc-Syk-ΔSH2-C, or Myc-Syk-ΔKD were lysed for an immunoprecipitation assay. Flag and Myc levels were measured by western blotting using whole cell lysates (input), or immunoprecipitants obtained from the whole cell lysates of HEK293 cells were measured by anti-Myc (IP: Myc). (g) HEK293 cells were transfected with Flag-TBK1-WT and Myc-Syk-WT, Myc-Syk-ΔSH2-N, Myc-Syk-ΔSH2-C, or Myc-Syk-ΔKD for 24 h and then permeabilized and fixed for a confocal analysis. Alexa Fluor 488 and 568 were used to recognize TBK1 (green) and Syk (red), respectively. (h) Phosphorylated and total Syk and Flag levels were measured by western blotting of whole cell lysates transfected with Flag-TBK1-WT, Flag-TBK1-ΔCC, Flag-TBK1-ΔULD, or Flag-TBK1-ΔKD. (i) An in vitro kinase assay was conducted with immunoprecipitated Syk or TBK1 proteins prepared from HEK293 cells transfected with Flag-TBK1-WT, Flag-TBK1-ΔCC, Flag-TBK1-ΔULD, or Flag-TBK1-ΔKD as enzymes and with immunoprecipitated p85 proteins prepared from Myc-p85-overexpressing HEK293 cells. Phosphorylated and total p85, Myc, and Flag levels were analyzed by western blotting. IgG, immunoglobulin G.

We next investigated the molecular interactions between TBK1 and Syk during inflammatory responses. Initially, we examined the binding interactions between TBK1 and Syk. TBK1-WT and Syk interacted directly in HEK293 cells, but TBK1-K38A failed to manifest any interaction with Syk (Figure 5a). To identify the specific interaction sites, domain deletion mutants of TBK1 were generated, and interactions were assessed by cotransfecting HEK293 cells with the appropriate mutant plasmids. Syk interacted with TBK1-WT and TBK1-ΔULD but not with TBK1-ΔCC and TBK1-KD (Figure 5c). Those results were confirmed by confocal microscopy of HEK293 cells transfected with TBK1-WT or the mutant constructs and Syk. TBK1-WT, TBK1-ΔULD, and Syk-WT had overlapping localization in HEK293 cells, but the colocalized region with Syk was reduced by TBK1-ΔCC and TBK1-K38A (Figure 5d). Next, to find the binding site of TBK1 on Syk, we used immunoprecipitation and confocal microscopy to observe alterations in their interaction when using domain deletion mutant plasmids of Syk (Figure 5e). TBK1-WT interacted with Syk-WT, Syk-ΔSH2-N, and Syk-ΔKD but not with Syk-ΔSH2-C (Figure 5f). Additionally, colocalization of TBK1 and Syk was notably suppressed in Syk-ΔSH2-C-transfected HEK293 cells (Figure 5g). The phosphorylation of Syk and the resulting kinase activity were examined in HEK293 cells transfected with TBK1 domain-deletion mutants. Although TBK1-WT and TBK1-ΔULD induced Syk phosphorylation, TBK1-K38A and TBK1-ΔCC neither induced nor reduced Syk phosphorylation (Figure 5h). Also, the increased kinase activity of Syk induced by TBK1-WT and TBK1-ΔULD was reduced in HEK293 cells transfected with TBK1-K38A or TBK1-ΔCC (Figure 5i). These results suggest that Syk is a key regulator of the MyD88-/TBK1-dependent NF-*κ*B activation pathway during inflammatory responses.

## 4. Discussion

TBK1, a multifaceted protein kinase, regulates a variety of cellular processes, including immune responses, autophagy, and cell survival and proliferation [6, 22, 29–31]. Previous reports have elucidated the role of Syk as an upstream regulator of TBK1. Within the TLR4-dependent signaling pathway, Syk modulates the interferon signaling pathway using TBK1 as a direct substrate. Moreover, Syk impairs the activation of TBK1 in lung cancer cells infected with influenza A virus [32, 33]. However, the role of TBK1 in NF-*κ*B activation signaling is not well-known, despite the well-documented importance of both TBK1 and NF-*κ*B in immune regulation [34]. This research addresses this knowledge gap by uncovering an intricate regulatory relationship between TBK1 and Syk in MyD88-dependent NF-*κ*B activation but not in the TRIF-dependent pathway. MyD88 is a pivotal adapter protein in innate immune responses [35] and a key mediator in the signaling pathways of TLRs and interleukin-1 receptors [35–37]. Upon ligand binding, MyD88 orchestrates downstream signaling cascades, culminating in the activation of NF-*κ*B and mitogen-activated protein kinases (MAPKs) [36, 37]. However, previous work has not fully elucidated the MyD88/TBK1/NF-*κ*B pathway.

In this study, we confirmed that TBK1 plays a critical role in TLR signaling events. We revealed that TBK1 phosphorylation is induced by the LPS, poly I:C, and Pam3CSK ligands of TLR4, TLR3, and TLR2, respectively (Figure 1a). TBK1 overexpression increased the expression of cytokines (IFNB1, TNF, NOS2, and IL1B) (Figure 1c) and enhanced luciferase activity mediated by NF-*κ*B (Figure 1d). Our results (Figure 2) showed that TBK1 activity is essential for the expression of TNF, NOS2, and IL1B via NF-*κ*B activation only under MyD88 activation conditions and not under TRIF activation conditions, as reported previously by Zhou et al. [38]. They observed that TBK1 is activated by MyD88 but not TRIF during LPS stimulation [38]. In contrast, the expression of IFNB1 seemed to require IRF3 activated by TBK1 under TRIF conditions [39], which agreed with our results following siTBK1 treatment of HEK293 cells stimulated by TRIF (Figure 2d,f). Therefore, TBK1 plays important roles in both NF-*κ*B and IRF3 activation via TLR signaling. Because both NF-*κ*B and IRF3 are important transcription factors in the innate immune responses of macrophages and dendritic cells [6, 40, 41], TBK1 appeared to be a critical player in innate immunity. Indeed, TBK1 knockout or deletion in mice and cells suppressed defense mechanisms against bacterial and viral infection [42, 43].

Syk, Src, and p85 are key regulators of the NF-*κ*B activation pathway [44–46]. In addition, MAPKs such as p38, JNK, and ERK are induced by MyD88 and JAK and are regarded as important molecules in both inflammatory responses and NF-*κ*B regulation [47, 48]. To identify the main downstream molecule(s) of TBK1, we use specific inhibitors. Inhibitors of Syk, p85, and NF-*κ*B suppress the mRNA expression of TNF and the luciferase activity of NF-*κ*B (Figure 3a, b). Moreover, phosphorylation of Syk, p85, and IκB*α* is reduced by TBK1 inhibition in MyD88-, TRIF-, and LPS-stimulated conditions (Figure 3c,d). Syk is already known to be upstream of p85 [49, 50]. We observe that inhibiting Syk with BAY-61-3606 reduces the phosphorylation of Syk and IκB*α* in RAW264.7 cells overexpressing TBK1 without altering the phosphorylation of TBK1 (Figure 3e). Although Syk is a critical mediator of TBK1-dependent IRF3 signaling [32, 33], no previous study has identified Syk activation by TBK1 in the NF-*κ*B pathway. Additionally, Syk phosphorylation can be induced via a MyD88-dependent pathway (Figure 4a). Silencing Syk in TBK1-overexpressing HEK293 cells inhibits the phosphorylation of IκB*α* and NF-*κ*B luciferase activity, but silencing TBK1 in Syk-overexpression conditions does not suppress the levels of p-Src and p-IκB*α* or the activity of NF-*κ*B-mediated luciferase (Figure 4c,d). In agreement with those data, the kinase activity of Syk decreases with siTBK1 transfection in Flag-MyD88 overexpression conditions (Figure 4f left panel). Our findings strongly imply that Syk can act as an intermediary to facilitate crosstalk between MyD88/TBK1 and p85/PI3K, leading to the NF-*κ*B pathway.

Finally, we used immunoprecipitation, immunoblotting analyses, and confocal microscopy with domain deletion mutants of TBK1 and Syk to examine whether TBK1 and Syk interact directly (Figure 5). Previously, the direct interaction of Syk and TBK1 was reported with whole proteins [32]. Interestingly, we found that the CC domain in TBK1 and the c-term SH2 domain in Syk are critical to the interaction of TBK1 and Syk. Importantly, TBK1 kinase activity seemed to be essential for Syk binding because the kinase deletion mutant of TBK1 showed reduced binding to Syk (Figure 5c,d). In contrast, the kinase domain of Syk did not affect its association with TBK1, implying that the enzyme activity of Src is not necessary for its molecular interaction with TBK1. Instead, because the kinase domain of Syk is crucial to the phosphorylation of substrates such as p85, a regulatory domain of PI3K [51], we suggest that Syk activated by TBK1 might manage PI3K-induced inflammatory responses. In conditions of increased TBK1 activity, strongly upregulated Syk might also be involved in inflammatory diseases such as neuroinflammation [52], rheumatoid arthritis [53], and lung inflammation [54]. Therefore, our results could indicate that the interaction between TBK1 and Syk is domain-specific, with the TBK1-mediated phosphorylation of Syk allowing the interaction between Syk and its substrates in the kinase domain. It was also reported that S510, S527, and S716 in the CC1 and CC2 domains are phosphorylated by AKT1, DYRK2, and PKCθ and that K670 is ubiquitinated by DTX4 [7], which implies that the CC domains are important to interactions with those proteins and to modulating the stability of TBK1. Therefore, the interaction between Syk and TBK1 in the CC domains could raise the possibility of regulating the interactions between TBK1 and AKT1, DYRK2, or PKC, as well as the protein stability of TBK1. We plan to examine that possibility in our future studies.

While our findings provide important insights into the role of TBK1 in immune signaling, there are several limitations. First, our experiments primarily focused on in vitro models, which are informative but may not fully capture the complexity of TBK1-Syk interactions in vivo. Further studies using animal models or patient-derived cells are necessary to validate our findings and assess the broader physiological relevance of the TBK1-Syk interaction in regulating inflammation. Additionally, whether other kinases or signaling proteins are involved in modulating TBK1 activity in different contexts needs to be investigated, particularly in diseases in which NF-*κ*B signaling is aberrantly activated.

**Figure 6:**
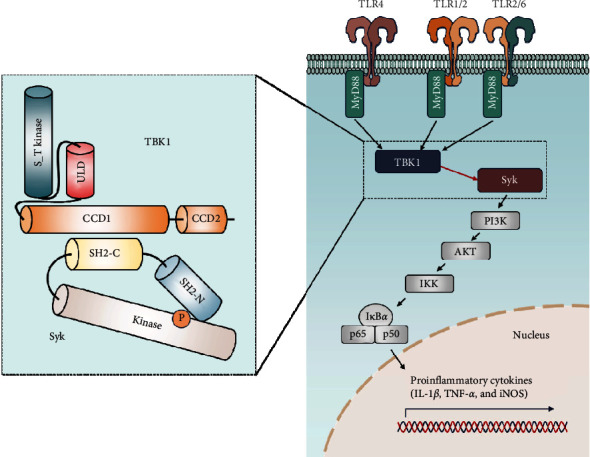
Schematic summary demonstrating the role of the TANK-binding kinase 1 (TBK1)–spleen tyrosine kinase (Syk) axis in inflammatory responses. Domain organization and interactions of TBK1 and Syk are shown including the serine–threonine kinase domain (S_T kinase), the ubiquitin-like domain (ULD), coiled coil domain 1 (CCD1), coiled coil domain 2 (CCD2), SRC homology domain 2 N-terminal (ΔSH2-N), SRC homology domain 2 C-terminal (ΔSH2-C), and kinase domain (ΔKD). Phosphorylated sites of Syk are indicated with P (orange).

In conclusion, our research suggested that TBK1 is essential for the activation of Syk in MyD88-mediated TLR pathways, and this regulation is driven by the direct interaction between TBK1 and Syk (Figure 6). By identifying Syk as a critical mediator in the TBK1-mediated NF-*κ*B pathway, we furthered our understanding of the intricate dynamics of immune signaling. This novel insight into the crosstalk between TBK1 and Syk not only enhances our fundamental understanding of inflammatory regulation but also presents promising therapeutic opportunities. The ability to modulate inflammatory responses through targeted manipulation of the TBK1-Syk interaction holds considerable potential for the development of new treatments for inflammation related diseases, representing a pivotal advancement in the search for more effective therapeutic strategies.

## Figures and Tables

**Table 1:** Primer sequences used for quantitative real-time polymerase chain reaction (PCR) and gene cloning in this study.

Target	Direction	Sequence (5′ to 3′)
Primers for real-time PCR for measuring *Mus musculus* genes		

IFNB1	Forward	AAGAGTTACACTGCCTTTGCCATC
	Reverse	CACTGTCTGCTGGTGGAGTTCATC

TNF	Forward	TGCCTATGTCTCAGCCTCTT
	Reverse	GAGGCCATTTGGGAACTTCT

NOS2	Forward	GGAGCCTTTAGACCTCAACAGA
	Reverse	TGAACGAGGAGGGTGGTG

IL1B	Forward	GGCCTTGGGCCTCAAAGGAA
	Reverse	GCTTGGGATCCACACTCTCCA

GAPDH	Forward	CAATGAATACGGCTACAGCAAC
	Reverse	AGGGAGATGCTCAGTGTTGG

Primers for real-time PCR for measuring *Homo sapiens* genes		

IFNB1	Forward	AAACTCATGAGCAGTCTGCA
	Reverse	AGGAGATCTTCAGTTTCGGAGG

TNF	Forward	GGCCCAGGCAGTCAGATCAT
	Reverse	TCTCTCAGCTCCACGCCATT

NOS2	Forward	CGCATGACCTTGGTGTTTGG
	Reverse	CATAGACCTTGGGCTTGCCA

IL1B	Forward	TGGACCTCTGCCCTCTGGAT
	Reverse	AAGGTCTGTGGGCAGGGAAC

GAPDH	Forward	CGGGAAACTGTGGCGTGATG
	Reverse	ATGACCTTGCCCACAGCCTT

Primers for cloning genes		

Syk-WT	Forward	CTCGAGAAGCCAGCAGCGGCATGG
	Reverse	CGGCCGTTAGTTCACCACGTCATAGTAGTAATTGCG

Syk-ΔSH2-1	Forward	CGGCCCCAAGGGGTGCAGC
	Reverse	GAAGGGCAGGTGGTTGGCG

Syk-ΔSH2-2	Forward	CAAAAAATCGGCACACAGGGA
	Reverse	AGGCATTTTTTCATGGGCTGT

Syk-ΔKD-1	Forward	GACGTGGTGAACTAACGGCCG
	Reverse	CAGCAGCTTTCGGTCCAGGT

TBK1-WT	Forward	GAGCTCGGATATGCAGAGCACTTCTAATCAT
	Reverse	CAACGTTGACTGTCTTTAGCTCGAGCATGC

TBK1-ΔKD	Forward	GCAGAAACTAGTGATATACTTCA
	Reverse	CAGATGATTAGAAGTGCTCTG

TBK1-ΔULD	Forward	CGGGAACCTCTGAATACCATA
	Reverse	ATCACTAGTTTCTGCAAAAAA

TBK1-ΔCC	Forward	TAGCTCGAGCATGCATCTAGA
	Reverse	CCGGCTTACTACAAATATAGGG

TBK1-K38A	Forward	AAAACTGGTGATTTATTTGCTATCGCAGTATTTAATAACATAAGCTTCCT
	Reverse	AGGAAGCTTATGTTATTAAATACTGCGATAGCAAATAAATCACCAGTTTT

## Data Availability

All data are available from the corresponding author.
